# Purification, conformational analysis and cytotoxic activities of host-defense peptides from the Tungara frog *Engystomops pustulosus* (Leptodactylidae; Leiuperinae)

**DOI:** 10.1007/s00726-023-03312-2

**Published:** 2023-08-07

**Authors:** J. Michael Conlon, Laure Guilhaudis, Samir Attoub, Laurent Coquet, Jérôme Leprince, Thierry Jouenne, Milena Mechkarska

**Affiliations:** 1 Diabetes Research Centre, School of Biomedical Sciences, Ulster University, Coleraine, BT52 1SA Northern Ireland UK; 2 Laboratoire COBRA (UMR 6014 & FR 3038), INSA de Rouen, CNRS, Université Rouen Normandie, 76000 Rouen, France; 3 Department of Pharmacology and Therapeutics, College of Medicine and Health Sciences, United Arab Emirates University, 17666 Al Ain, United Arab Emirates; 4 CNRS UAR2026, HeRacLeS-PISSARO PBS UMR 6270, Université Rouen Normandie, 76000 Rouen, France; 5 INSERM, Normandie Université, NorDiC UMR 1239, HeRacLeS, US 51, PRIMACEN, Université Rouen Normandie, 76000 Rouen, France; 6 Department of Life Sciences, Faculty of Science and Technology, The University of The West Indies, St. Augustine Campus, St. Augustine, Trinidad and Tobago

**Keywords:** Host-defense peptide, Cytotoxic, Frog skin, Pustulosin, Tigerinin

## Abstract

The amphibian family Leptodactylidae is divided into three sub-families: Leiuperinae, Leptodactylinae, and Paratelmatobiinae. Host-defense peptides (HDPs) present in the skins of frogs belonging to the Leptodactylinae have been studied extensively, but information is limited  regarding peptides from Leiuperinae species. Peptidomic analysis of norepinephrine-stimulated skin secretions from the Tungara frog *Engystomops pustulosus* (Leiuperinae) collected in Trinidad led to the isolation and structural characterization of previously undescribed pustulosin-1 (FWKADVKEIG KKLAAKLAEELAKKLGEQ), [Q28E] pustulosin-1 (pustulosin-2), and pustulosin-3 (DWKETAKELLKKIGAKVAQVISDKLNPAPQ). The primary structures of these peptides do not resemble those of previously described frog skin HDPs. In addition, the secretions contained tigerinin-1EP (GCKTYLIEPPVCT) with structural similarity to the tigerinins previously identified in skin secretions from frogs from the family Dicroglossidae. Pustulosin-1 and -3 adopted extended α-helical conformations in 25% trifluoroethanol–water and in the presence of cell membrane models (sodium dodecylsulfate and dodecylphosphocholine micelles). Pustulosin-1 and -3 displayed cytotoxic activity against a range of human tumor-derived cell lines (A549, MDA-MB-231, and HT29), but their therapeutic potential for development into anti-cancer agents is limited by their comparable cytotoxic activity against non-neoplastic human umbilical vein endothelial cells. The peptides also displayed weak antimicrobial activity against *Escherichia coli* (MIC = 125 µM) but were inactive against *Staphylococcus aureus.* Tigerinin-1EP was inactive against both the tumor-derived cells and bacteria.

**Supplementary Information:**

The online version contains supplementary material available at 10.1007/s00726-023-03312-2.

## Introduction

The amphibian family Leptodactylidae, currently comprising 232 recognized species, is divided into three sub-families—Leiuperinae (101 species), Leptodactylinae (117 species), and Paratelmatobiinae (14 species) (Frost 2023). Within the sub-family Leiuperinae, the genus *Engystomops*, sometimes referred to as foam frogs (Hedges et al. 2019), comprises nine species. The Tungara frog *Engystomops pustulosus* (formerly *Physalaemus pustulosus*) is a small (snout-to-vent length between 25 and 35 mm) nocturnal species that adapts well to a range of environments. It is distributed in eastern and southern Mexico, south and east through Central America to Colombia, Venezuela, and Guyana with a population in Trinidad and Tobago (Weigt et al. 2005; Murphy et al. 2018; Frost 2023). However, evidence derived from analysis of mitochondrial DNA suggests significant genetic divergence between lineages across this wide range (Pröhl et al. 2010). The frog is listed by the International Union for Conservation of Nature’s Red List of Threatened Species as a species of least concern (IUCN Amphibian Specialist Group 2020), but it has been observed that pollutants at agricultural sites have resulted in decreased egg numbers, reduced hatching success, and undersized/smaller body size and male secondary sexual characteristics which may lead to population declines (Orton et al. 2022). The species is also susceptible to the emergent infectious disease chytridiomycosis caused by the fungal pathogen *Batrachochytrium dendrobatidis* that is contributing to worldwide amphibian population declines (Rodríguez-Brenes et al. 2016).

The Tungara frog is best known for the ability of the male to produce an extremely stable bio-foam nest to provide a protective environment for fertilized eggs and larvae. The nest serves as a defense against pathogenic microorganisms, parasites, and predators, prevents desiccation, and reduces UV damage (Brozio et al. 2021). The foam contains a mixture of six proteins, termed ranaspumins (Rsn-1 to Rsn-6), in major abundance. Rsn-3 to Rsn-6 are lectins, and Rsn-2 exhibits substantial detergent-like surfactant activity necessary for production of foam and Rsn-1 is structurally similar to proteinase inhibitors of the cystatin class (Fleming et al. 2009).

Ever since the pioneering studies of the group of Erspamer from the 1960s (Anastasi et al. 1964), it has been known that skin secretions from a range of frog species contain high concentrations of peptides with a diverse spectrum of biological activities (Xu and Lai 2015; Conlon et al. 2019). Of particular note are host-defense peptides (HDPs) with varying degrees of ability to inhibit the growth of clinically relevant pathogenic bacteria and fungi that are believed to be a component of the frog’s system of innate immunity (Varga et al. 2019). They may also possess the ability to permeabilize mammalian cells and act synergistically with toxins in the secretions to deter ingestion by predators (Raaymakers et al. 2017). The present study describes the purification, structural characterization, conformational analysis, and cytotoxic activities of three structurally related HDPs, termed pustulosin-1, -2, and -3, from norepinephrine-stimulated skin secretions of *E. pustulosus* whose amino acid sequences do not resemble those of previously described frog skin peptides together with a peptide, termed tigerinin-1EP, with structural similarity to the tigerinins previously identified in skin secretions of frogs from the family Dicroglossidae.

## Materials and methods

### Collection of skin secretions

Adult *E. pustulosus* (*n* = 10, sex not determined) were collected at Waterville Estate, Santa Cruz, Trinidad in March 2022. The animals were sampled in the field and subsequently released unharmed at the site of capture. Each frog was injected via the dorsal lymph sac with norepinephrine hydrochloride (40 nmol/g body weight) and placed in distilled water (100 mL) for 15 min. The collection solution was acidified by addition of concentrated hydrochloric acid (0.5 mL) and immediately frozen.

### Purification of the peptides

The solutions containing the secretions were pooled and passed at a flow rate of approximately 2 mL.min^−1^ through 6 Sep-Pak C-18 cartridges (Waters Associates, Milford, MA, USA) connected in series. Bound material was eluted with acetonitrile/water/trifluoroacetic acid (TFA) (70.0:29.9:0.1, v/v/v) and freeze-dried. The material was redissolved in 0.1% (*v*/*v*) TFA/water (2 mL) and injected onto a semipreparative (1.0 cm × 25 cm) Vydac 218TP510 (C-18) reversed-phase HPLC column (Grace, Deerfield, IL, USA) equilibrated with 0.1% (*v*/*v*) TFA/water at a flow rate of 2.0 mL.min^−1^. The concentration of acetonitrile in the eluting solvent was raised to 21% (*v*/*v*) over 10 min and to 63% (*v*/*v*) over 60 min using linear gradients. Absorbance was monitored at 214 nm and fractions (1 min) were collected using a BioRad 2110 fraction collector. The peptides within the peaks designated 1–4 that were present in major abundance were subjected to further purification by successive chromatographies on (1.0 cm × 25 cm) Vydac 214TP510 (C-4) and (1.0 cm × 25 cm) Vydac 208TP510 (C-8) columns. The concentration of acetonitrile in the eluting solvent was raised from 21 to 56% (*v*/*v*) over 50 min for peak 1 and from 28 to 63% for peaks 2–4. The flow rate was 2.0 mL.min^−1^ and fractions were collected by hand.

### Structural characterization

MALDI-TOF mass spectrometry was carried out using an UltrafleXtreme instrument (Bruker Daltonik, Bremen, Germany). Full details of the procedure, including calibration of the instrument with peptides of known molecular mass in the 1–4 kDa range, have been provided previously (Conlon et al. 2018). The accuracy of mass determinations was < 0.02%. The primary structures of the purified peptides were determined by automated Edman degradation using an Applied Biosystems model 494 Procise sequenator (Applied Biosystems, Courtaboeuf, France).

### Peptide synthesis

Tigerinin-1EP, pustulosin-1, and pustulosin-3 were supplied in crude form by PEPMIC (Suzhou, China) and were purified to near homogeneity (> 98% purity) by reversed-phase HPLC on a (2.2 cm × 25 cm) Vydac 218TP1022 (C-18) column equilibrated with acetonitrile/water/TFA (35.0/64.9.9.9/0.1, v/v/v) at a flow rate of 6 mL.min^−1^. The concentration of acetonitrile was raised to 63% (*v*/*v*) over 60 min using a linear gradient. Absorbance was measured at 214 nm and the major peak in the chromatogram was collected manually. The identities of the peptides were confirmed by electrospray mass spectrometry.

### Conformational analysis

Secondary structure predictions were obtained using the AGADIR program which predicts the helical behavior of monomeric peptides based on the helix/coil transition theory (Muñoz and Serrano 1994). Physicochemical characteristics of pustulosin-1 and pustulosin-3 were calculated using the peptide analysis tools available on the HELIQUEST server (Gautier et al. 2008). Circular dichroism spectra were obtained using a MOS-500 CD spectrometer (BioLogic, Seyssinet-Pariset, France) as previously described (Pantic et al. 2019). Pustulosin-1 and pustulosin-3 were dissolved in (A) water, (B) 2,2,2-trifluoroethanol (TFE)–water (25% and 50%, *v*/*v*), (C) 10 mM sodium dodecyl sulfate (SDS) aqueous solution, and (D) 10 mM dodecylphosphocholine (DPC) aqueous solution at a final concentration of 0.25 mg mL^−1^. Three scans were accumulated and averaged for each sample. All spectra were corrected by subtraction of the background obtained for each peptide-free solution. Circular dichroism measurements are reported as mean residue molar ellipticity ([θ]_MRE_ (deg cm^2^ dmol^−1^). Peptide secondary structure was estimated using the online CD spectra deconvolution server Dichroweb (Whitmore and Wallace 2004; 2008; Miles et al. 2022). For Dichroweb analysis, the secondary structure content was obtained by averaging the results given by CONTINLL (Provencher and Glockner 1981; Van Stokkum et al. 1990), CDSSTR (Compton and Johnson 1986; Sreerama and Woody 2000), and SELCON3 (Sreerama and Woody 1993, Sreerema et al. 1999) deconvolution programs. The α-helical content was also calculated using the Forood formula: 100 × ([θ]_222_/max[θ]_222_) with max[θ]_222_ =  − 40,000 [1 − (2.5/n)], where *n* = number of amino acid residues (Forood et al. 1993).

### Antimicrobial and cytotoxicity assays

Reference strains of microorganisms were purchased from the American Type Culture Collection (Rockville, MD, USA). Minimum inhibitory concentration (MIC) of synthetic pustulosin-1, pustulosin-3, and tigerinin-1EP against a clinically relevant Gram-positive bacterium, ampicillin-resistant *Staphylococcus aureus* (ATCC 12600), and a clinically relevant Gram-negative bacterium, *Escherichia coli* (ATCC 35218) were measured by the standard microdilution method mandated by the Clinical Laboratory and Standards Institute (CLSI 2018) as previously described (Barran et al. 2020). Control incubations were carried out in parallel with increasing concentrations of vancomycin for *S. aureus* and ampicillin for *E. coli* to monitor the validity and reproducibility of the assays.

Cytotoxicities against A549 human non-small cell lung adenocarcinoma cells (RRID:CVCL_0023), MDA-MB-231 human breast adenocarcinoma cells (RRID:CVCL_0062), HT-29 human colorectal adenocarcinoma cells (RRID:CVCL_0320), and human umbilical vein endothelial cells (HUVEC) (RRID:CVCL_2959) were measured as previously described (Manzo et al. 2015). The effects of the peptides (1–100 μM) on cell viability following a 24 h incubation were determined by measurement of ATP concentrations using a CellTiter-Glo Luminescent Cell Viability assay (Promega Corporation, Madison, WI, USA). The LC_50_ value was taken as the mean concentration of peptide producing 50% cell death in a minimum of three independent experiments.

Hemolytic activity of the peptides in the concentration range 37.5–300 µM against freshly prepared erythrocytes from male NIH male Swiss mice (Harlan Ltd, Bicester, UK) was determined as previously described (Barran et al. 2020). The LC_50_ value was taken as the mean concentration of peptide producing 50% hemolysis in three independent incubations.

## Results

### Purification of the peptides

**Fig. 1:**
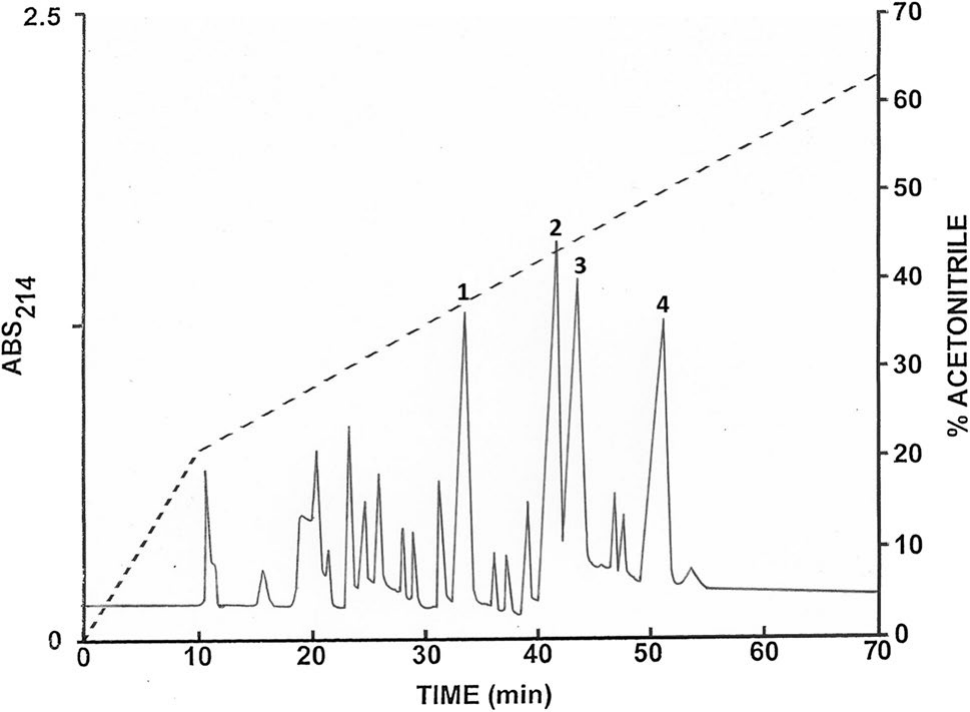
Reversed-phase HPLC on a semipreparative Vydac C-18 column of pooled skin secretions from ten *E. pustulosus* frogs collected in Trinidad after partial purification on Sep-Pak C-18 cartridges. The dashed line shows the concentration of acetonitrile in the eluting solvent. The peaks denoted 1–4 contain host-defense peptides that were purified further

**Fig. 2:**
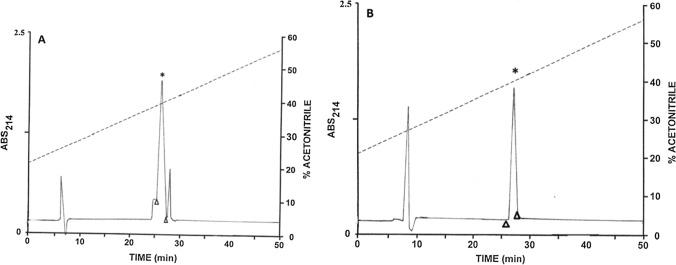
Purification to near homogeneity of tigerinin-1EP on **A** a semipreparative Vydac C-4 column and **B** a semipreparative Vydac C-8 column. The arrowheads show where peak collection began and ended. The dashed line shows the concentration of acetonitrile in the eluting solvent

The pooled skin secretions, after partial purification on Sep-Pak C-18 cartridges, were chromatographed on a Vydac C-18 semipreparative reversed-phase HPLC column (Fig. 1). The prominent peaks designated 1–4 were collected and subjected to further purification. Subsequent structural analysis showed that peak 1 contained tigerinin-1EP, peak 2 pustulosin-1, peak 3 pustulosin-2, and peak 4 pustulosin-3 together with a small amount (< 10%) of pustulosin-4. Pustulosin-1 and -2 and tigerinin-1EP were purified to near homogeneity (purity > 98% as assessed by a symmetrical peak shape, Edman degradation, and mass spectrometry) by further chromatography on semipreparative Vydac C-4 and Vydac C-8 columns. The methodology is illustrated by the purification of tigerinin-1EP on a Vydac C-4 column (Fig. 2A) and a Vydac C-8 column (Fig. 2B). Attempts to separate the very hydrophobic pustulosin-3 from the pustulosin-4 impurity by HPLC were not successful, and so, the peptide mixture was subjected to automated Edman degradation.

### Structural characterization

The primary structures of tigerinin-1EP, pustulosin-1, and pustulosin-2 were established without. Ambiguity by automated Edman degradation and their complete primary structures are shown in Table 1. The molecular masses of the peptides, determined by MALDI-TOF mass spectrometry, were consistent with the proposed structures and are also shown in Table 1. The data indicate that tigerinin-1EP was isolated in the oxidized (disulfide-bridged) form. Analysis of the pustulosin-3 isolate by mass spectrometry revealed the presence of a minor component of molecular mass [MH^+^] = 3274.6 in addition to the major component with [MH^+^] = 3318.7. During Edman degradation of the peptide mixture, additional phenylthiohydantoin derivatives in low abundance were detected during cycles corresponding to positions 4, 5, 8, 9, and 17 in the peptide. Single phenylthiohydantoin derivatives were detected during all other cycles. Although it was not possible to identify the different amino acids in the minor component with certainty, the most probable proposed sequence DWKADAKDILKKIGAKIAQVISDKLNPAPQ corresponds exactly with the C-terminal domain of a 71-amino-acid hypothetical protein GD081_021018 (Gen Bank Accession KAG8549497.1) predicted from the nucleotide sequence of an *E. pustulosus* gene. Consequently, the minor component present in the pustulosin-3 preparation is provisionally termed pustulosin-4. The observed molecular mass of the peptide is consistent with its proposed structure (Table 1). A protein–protein NCBI BLAST search (National Center for Biotechnology Information) indicated a lack of sequence identity of the pustulosins with HDPs from any other frog species in the database.

**Table 1:** Primary structures and molecular masses of the peptides isolated from norepinephrine-stimulated skin secretions from *E. pustulosus*

Peak no.	Peptide	Primary structure	[MH^+^]_exp_	[MH^+^]_calc_
1	Tigerinin-1EP	GCKTYLIEPPVCT	1421.7	1421.7
2	Pustulosin-1	FWKADVKEIGKKLAAKLAEELAKKLGEQ	3141.6	3141.8
3	Pustulosin-2	FWKADVKEIGKKLAAKLAEELAKKLGEE	3142.6	3142.8
4	Pustulosin-3	DWKETAKELLKKIGAKVAQVISDKLNPAPQ	3318.7	3318.9
4	Pustulosin-4*	DWKADAKDILKKIGAKIAQVISDKLNPAPQ	3274.6	3274.8

Peak number refers to Fig. 1. [MH^+^]_exp_ denotes the experimentally determined molecular mass and [MH^+^]_calc_ denotes the mass calculated from the proposed structures. *Pustulosin-4 was not obtained in pure form but was detected as an impurity in pustulosin-3

### Conformational analysis

**Fig. 3:**
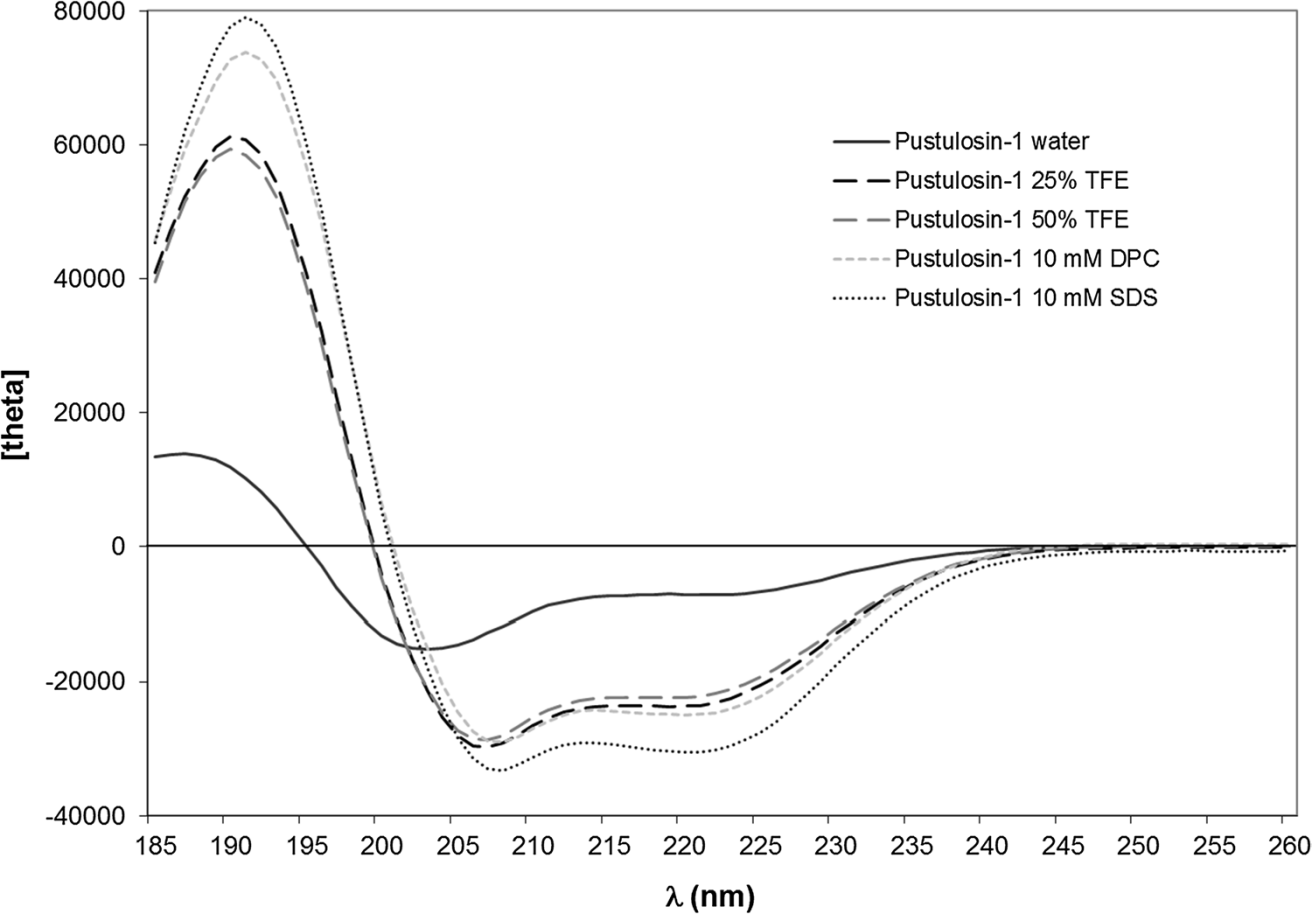
Circular dichroism spectra of pustulosin-1 at room temperature and at a concentration of 0.25 mg.mL.^−1^ in **A** water (black solid line), **B** 25% (*v*/*v*) TFE–water (black dashed line), **C** 50% (*v*/*v*) TFE–water (grey dashed line), **D** 10 mM dodecylphosphocholine (DPC) aqueous solution (grey dotted line), and **E** 10 mM sodium dodecyl sulfate (SDS) aqueous solution (black dotted line)

**Fig. 4:**
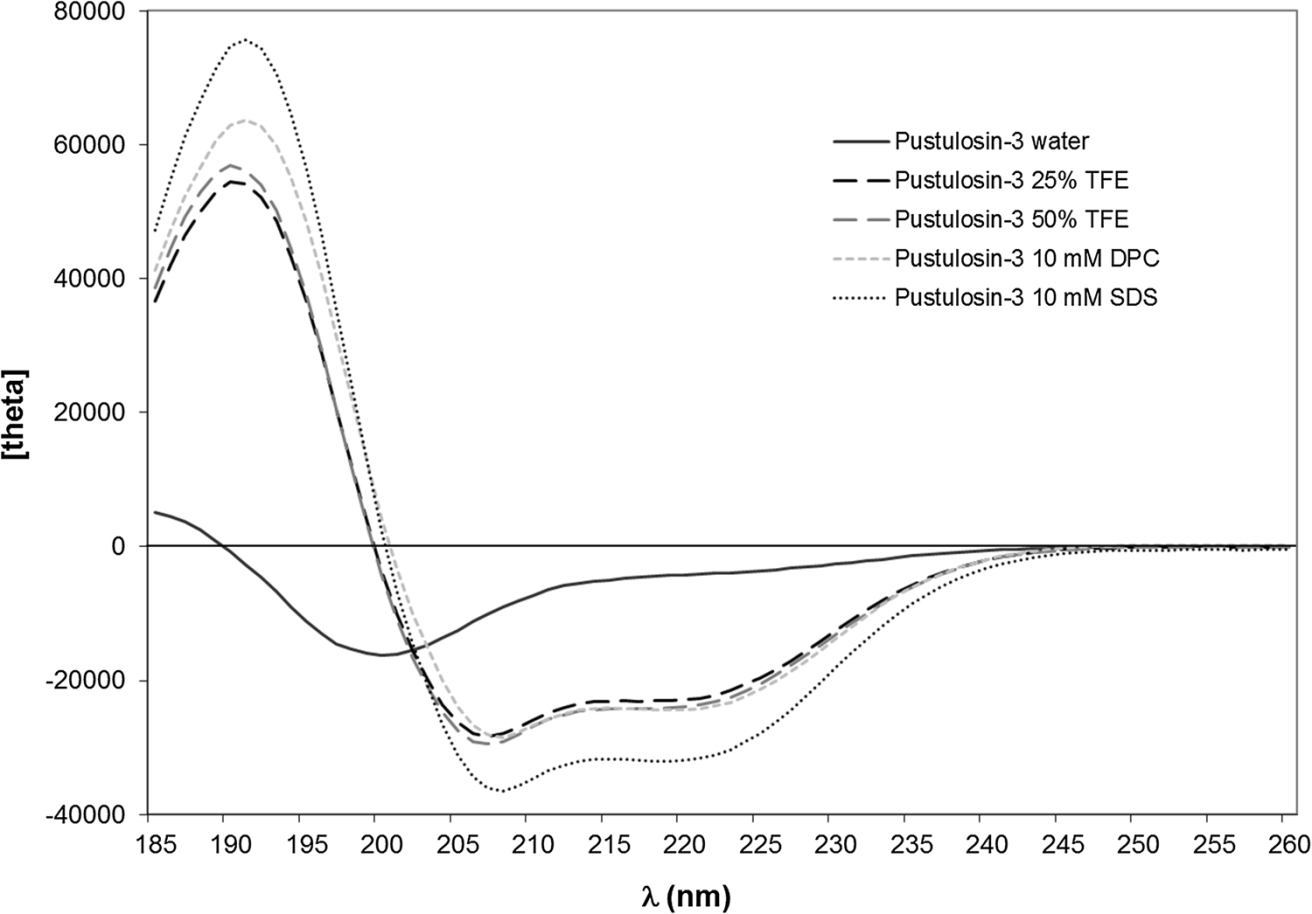
Circular dichroism spectra of pustulosin-3 at room temperature and at a concentration of 0.25 mg.mL.^−1^ in **A** water (black solid line), **B** 25% (*v*/*v*) TFE–water (black dashed line), **C** 50% (*v*/*v*) TFE–water) (grey dashed line), **D** 10 mM dodecylphosphocholine (DPC) aqueous solution (grey dotted line), and **E** 10 mM sodium dodecyl sulfate (SDS) aqueous solution (black dotted line)

The AGADIR program predicts that pustulosin-1 has a very strong propensity for adopting a α-helical conformation between residues 3 and 24, and pustulosin-3 has a similar very high probability of forming a stable α-helix between residues 2 and 26. This prediction is supported by measurement of circular dichroism spectra in a range of solvents. Figures 3 and 4 show the spectra of pustulosin-1 and pustulosin-3 in water, 25% TFE–water, 50% TFE–water, 10 mM SDS aqueous solution, and 10 mM DPC aqueous solution, respectively.

Both peptides adopt similar behavior. In water, far-UV CD spectra exhibited a small positive maximum around 187 nm (Figs. 3 and 4) characteristic of a low content of ordered conformation. For pustulosin-1, the presence of a minimum around 203 nm and a negative shoulder between 215 and 230 nm indicated the presence of a helical conformation. In agreement with the strong helical forming propensities of pustulosin-1 and -3, the addition of a relatively small amount of TFE, a solvent known for its ability to stabilize secondary structure, was sufficient to stabilize the helical conformation as shown by the similarity of the spectra recorded in the presence of 25% and 50% TFE (Figs. 3 and 4). In these media, CD spectra showed typical α-helical features with a positive peak at ~ 190 nm and double negative minima around 208 and 222 nm. The percentage of α-helix estimated from the mean residue ellipticity at 222 nm and of helical content using the Dichroweb server (Table 2) was around 65% for both peptides. This corresponds to about 18 residues out of 27 for pustulosin-1 and 20 out of 30 for pustulosin-3.

**Table 2:** Prediction of secondary structure content from CD spectra of pustulosin-1 and pustulosin-3

Peptide	Medium	Method	helix	β sheet	turns	random
Pustulosin-1	water	Dichroweb	21	14	14	52
		[Θ]_222_	20	–	–	–
	25% TFE	Dichroweb	71	7	3	18
		[Θ]_222_	63	–	–	–
	50% TFE	Dichroweb	66	5	7	22
		[Θ]_222_	60	–	–	–
	10 mM DPC	Dichroweb	83	2	2	14
		[Θ]_222_	68	–	–	–
	10 mM SDS	Dichroweb	86	1	3	12
		[Θ]_222_	83			
Pustulosin-3	water	Dichroweb	7	14	10	70
		[Θ]_222_	11	–	–	–
	25% TFE	Dichroweb	66	4	8	22
		[Θ]_222_	60	–	–	–
	50% TFE	Dichroweb	67	4	8	21
		[Θ]_222_	63	–	–	–
	10 mM DPC	Dichroweb	77	2	3	16
		[Θ]_222_	65	–	–	–
	10 mM SDS	Dichroweb	84	2	5	14
		[Θ]_222_	85	–	–	–

Values are given in percents

[Θ]_222_ corresponds to the % helicity calculated using the Forood formula (Forood et al. 1993). TFE 2,2,2-trifluoroethanol

*DPC* dodecylphosphocholine, *SDS* sodium dodecyl sulfate

In the presence of membrane mimics, the increase of the mean residue molar ellipticities’ values at 192 nm showed that the peptides were even more structured than in the presence of 25% of trifluoroethanol. When incubated with micelles composed of negatively charged surfactants (SDS), the two pustulosins exhibited 85% α-helical conformation. In the presence of zwitterionic micelles (DPC), the ellipticities’ values of the negative peaks of the α-helix (208 and 222 nm) were higher than in the presence of SDS suggesting a decrease in the helical content. Although the α-helical contents calculated by the Forood formula were lower (68% for pustulosin-1 and 65% for pustulosin-3), the analysis using Dichroweb indicated that both peptides contained a very high level of helical structure (83 and 77%, respectively). Such differences have been reported when peptide adopts helical conformations comprising non-canonical helical structures (Banerjee and Sheet 2017). Thus, in the presence of membrane mimics, pustulosin-1 contained a helix of about 23 residues and pustulosin-3 a helix ranging from 23 to 25 residues consistent with conformations predicted using the AGADIR program.

### Antimicrobial and cytotoxicity activities

Incubation of synthetic replicates of pustulosin-1 and pustulosin-3 for 24 h with a range of human tumor-derived cells and with HUVEC resulted in a decrease of cell viability determined by measurement of ATP concentrations. Table 3 displays the potencies of the peptides (LC_50_ values) revealing that pustulosin-3 exhibited greater cytotoxic activity than pustulosin-1 in all cases. The hemolytic activity of both peptides against mouse erythrocytes was low (LC_50_ > 300 µM) (Table 3). Tigerinin-1EP lacked cytotoxicity against the human cell lines at concentrations up to 100 µM and against erythrocytes at concentrations up to 300 µM.

**Table 3:** Cytotoxicities of peptides from *E. pustulosus* against lung adenocarcinoma A549 cells, breast adenocarcinoma MDA-MB-231 cells, colorectal adenocarcinoma HT-29 cells, human umbilical vein endothelial cells (HUVEC), and mouse red blood cells (RBC)

Cell	Pustulosin-1	Pustulosin-3	Tigerinin-1EP
A549	34 ± 1	9 ± 1	> 100
MDA-MB-231	73 ± 1	26 ± 1	> 100
HT-29	> 100 (41%)	50 ± 2	> 100
HUVEC	60 ± 2	17 ± 1	> 100
RBC	> 300 (17%)	> 300 (40%)	> 300 (0%)

Data show mean LC_50_ values (μM) ± S.E.M. The values in parentheses show the % hemolysis of mouse erythrocytes at 300 μM concentration for the three peptides and the % cell death of HT-29 cells for pustulosin-1 at 100 μM concentration

Pustulosin-1 and pustulosin-3 showed relatively weak growth inhibitory activity against a reference strain of the Gram-negative bacterium *E. coli* (MIC = 125 µM) but were inactive against an ampicillin-resistant strain of the Gram-positive bacterium *S. aureus* at concentrations up to 125 µM. The corresponding MIC value for ampicillin was 2.5 µg.mL^−1^ (*E. coli*) and 2.5 µg.mL^−1^ for vancomycin (*S. aureus*). A synthetic replicate of tigrerinin-1EP did not inhibit the growth of either *E. coli* or *S. aureus* at concentrations up to 125 µM.

## Discussion

The study has described the purification and structural characterization of HDPs in norepinephrine-stimulated skin secretion of *E. pustulosus*, a species belonging to the sub-family Leiuperinae within the family Leptodactylidae. Unlike frogs from the Leiuperinae, frogs from the sub-family Leptodactylinae have been investigated extensively for the presence of HDPs in skin secretions. Ocellatins, named after the first species, *Leptodactylus ocellatus* in which they were detected (Nascimento et al. 2004; Conlon 2008), have been isolated from a range of species belonging to the genus *Leptodactylus* [reviewed in (Barran et al. 2020)]. Additionally, members of conformationally flexible, glycine/leucine-rich plasticin family have been purified from skin secretions of *Leptodactylus pentadactylus* (Sousa et al. 2009) and *Leptodacylus laticeps* (Conlon et al. 2009). Plasticin-L1 from *L. laticeps* lacks antimicrobial activity but displays cytokine-mediated immunomodulatory properties stimulating production of the proinflammatory cytokines from murine peritoneal macrophages (Scorciapino et al. 2013). A comparison of the amino acid sequence of the pustulosins from *E. pustulosus* with the ocellatins of known structure (Supplementary Fig. 1) reveals very little sequence similarity. Among the ocellatins, only residues G1, D4, K7, K11, and K20 have been strongly conserved (Marani et al. 2020). Of these, only residues K7 and K11 are present in the pustulosins. The genus Pleurodema is also included in sub-family Leiuperinae (Frost 2023) and the structure of a glycine–leucine-rich antimicrobial peptide termed thaulin-1 (GNLLGGLLRPVLGVVKGLTGGL) has been predicted from the nucleotide sequence of a cDNA from a *Pleurodema thaul* skin library (Marani et al. 2017). In a related study, antimicrobial peptides termed somuncurin-1 (FIIWPLRYRK), somuncurin-2 (FILKRSYPQYY), and thaulin-3 (NLVGSLLGGILKK) were identified in the skin of the frog *Pleurodema somuncurense* (Cancelarich et al. 2020). As with the ocellatins, these peptides show little or no sequence similarity with the pustulosins.

Cationic α-helical peptides, including those isolated from frog skin secretions (Conlon et al. 2019), have been recognized as agents with therapeutic potential for development into anti-cancer agents (Chen et al. 2023). The ability of pustulosin-3 to produce death in vitro of non-small cell lung adenocarcinoma A549 cells, breast adenocarcinoma MDA-MB-231 cells, and colorectal adenocarcinoma HT-29 cells during a 24 h incubation (LC_50_ values in the range 9–50 µM) together with its low hemolytic activity against mouse erythrocytes (LC_50_ > 300 µM) is encouraging in this regard. However, in common with other cytotoxic peptides isolated from amphibians (Mechkarska et al. 2014; Serra et al. 2014; Conlon et al. 2023), the peptide shows little selectivity for cancer cells. The LC_50_ value against non-neoplastic human umbilical vein endothelial cells of pustulosin-3 was 17 µM (Table 3). Future studies will address the design of analogs of the pustulosins with improved potencies and specificities.

**Fig. 5:**
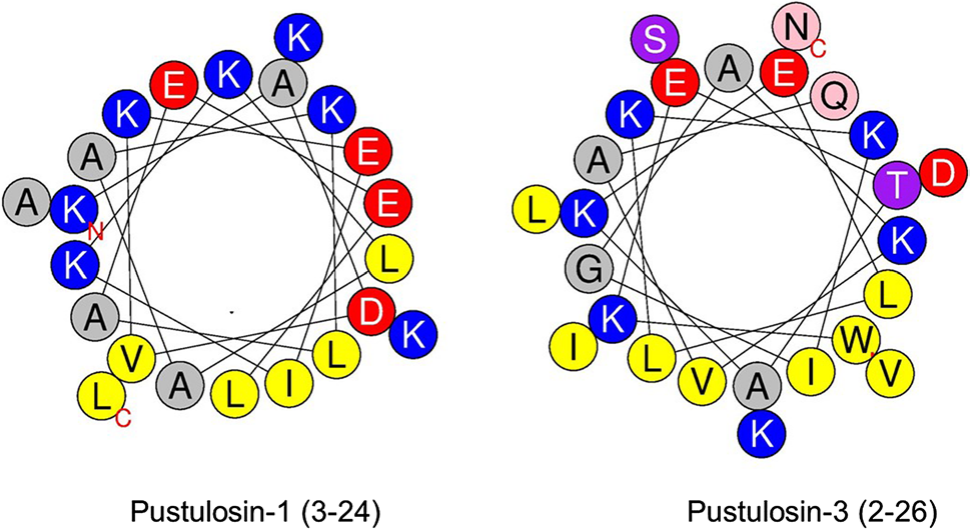
A Schiffer–Edmundson wheel representation of the predicted α-helical domains of pustulosin-1 and pustulosin-3. Basic amino acids are shown in blue, acidic amino acids in red, and strongly hydrophobic amino acids in yellow

Like the pustulosins investigated in this study, most ocellatins studied to-date exhibit relatively weak antimicrobial activity (minimum inhibitory concentration, MIC ≥ 50 µM) against Gram-negative bacteria and are inactive (MIC > 100 µM) against Gram-positive bacteria. Ocellatin-4 from *L. ocellatus* (Nascimento et al. 2007) and ocellatin-3N from *Leptodactylus nesiotus* (Barran et al. 2020) are exceptions to this generalization displaying broad-spectrum antimicrobial activity as well as moderately high hemolytic activity. With very few exceptions, frog skin HDPs, including the ocellatins (Gomes et al. 2018), are cationic (molecular charge at pH 7 between + 1 and + 5), contain a high proportion of hydrophobic residues, and adopt an amphipathic α-helical conformation in a membrane mimetic environment (Kabelka and Vácha 2021). Pustulosin-1 and -3 exhibit a molecular charge of + 2 at pH 7 and, as shown in Fig. 5, Schiffer–Edmundson wheel representations of the predicted helical regions of the peptides (Schiffer and Edmundson 1967) constructed using the HeliQuest web-server (Gautier et al. 2008) indicate that both peptides are associated with a hydrophobic domain comprising several Leu, Ile, and Val residues that facilitates binding to (phospho)lipids in the bacterial cell membrane and an extensive hydrophilic domain comprising multiple Lys, Glu, and Asp residues that promotes loss of integrity of the membrane (Vineeth Kumar and Sanil 2017). The occurrence of so many negatively charged amino acids in the > 1000 amphibian HDPs listed in the APD3 Antimicrobial Peptide Database (Wang et al. 2016) is unusual. The presence of these Glu and Asp residues in pustulosin-1 and -3 may account for relatively weak growth inhibitory activity of the peptides against the bacteria tested. The bacterial cell membrane is rich in anionic phospholipids, such as phosphatidylglycerol, and negatively charged lipopolysaccharides (Strahl and Errington 2017), so that the presence of multiple Glu and Asp residues in the peptides may inhibit binding and/or insertion into the membrane. Nevertheless, the pustulosins, possibly acting in concert with other toxic compounds in the skin secretions, may at least contribute to the observed antimicrobial activity of the bio-foam used by the male frogs to nest build (Brozio et al. 2021). The possibility remains open that the pustulosins play additional roles such an involvement in the reproductive strategy of the frog as in the case of the peptide pheromone splendipherin produced in the skin of *Litoria splendida* (Wabnitz et al. 2000) and Leptodactylus Aggression Stimulating Peptide (LASP) present in skin secretions of *Leptodactylus fallax* that provokes male–male aggressive interactions at the onset of the breeding season (King et al. 2005).

**Fig. 6:**
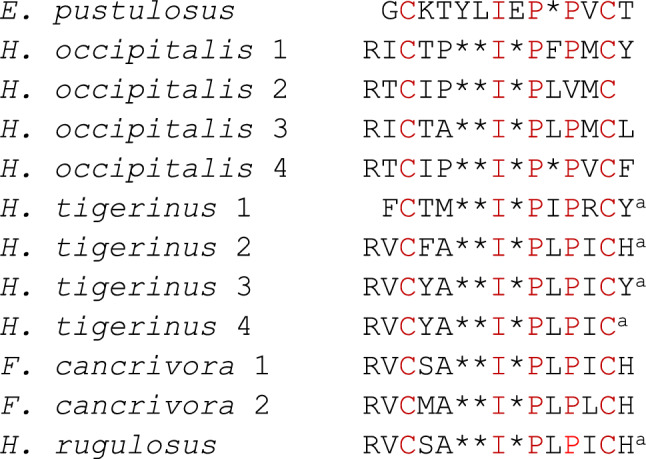
A comparison of the primary structure of tigerinin-1EP from *E. pustulosus* with tigerinins isolated from frogs belonging to the family Dicroglossidae. Conserved residues are shown in red. Gaps denoted by * are inserted into the sequences to maximize sequence similarity

The term tigerinin refers to a group of small (11–13 amino acid residues), structurally related peptides that contain an intramolecular disulfide bond and two or more proline residues. The tigerinins were first identified in the Indian frog *Hoplobatrachus tigerinus* (formerly *Rana tigerina*) (Sai et al. 2001) in the family Dicroglossidae but have subsequently been isolated from skin secretions of *Hoplobatrachus rugulosus* (Ojo et al. 2011), *Hoplobatrachus occipitalis* (McLaughlin et al. 2016), and *Fejervarya cancrivora* (Song et al. 2009) also in the family Dicroglossidae. The primary structures of these peptides are compared with tigerinin-1EP in Fig. 6. In common with tigerinin-1EP, most tigerinins studied to-date lack antimicrobial and hemolytic activities but incubation of tigerinin-1R from *H. rugulosus* with murine peritoneal macrophages and human peripheral blood mononuclear cells increased production of the anti-inflammatory cytokine IL-10 (Pantic et al. 2014). In addition, tigerinin-1R (Ojo et al. 2011) and tigerinin-1O from *H. occipitalis* (McLaughlin et al. 2016) potently stimulated the release of insulin from BRIN-BD11 clonal β-cells and tigerinin-4O stimulated the release of glucagon-like peptide-1 from GLUTag enteroendocrine cells (McLaughlin et al. 2016). Clearly, further studies are warranted to assess the therapeutic potential of tigerinin-1EP as an immunomodulatory agent in patients with endotoxemic complications such as severe sepsis and septic shock and/or as an antidiabetic agent in patients with Type 2 diabetes.

## Conclusion

The study has expanded our knowledge of the naturally occurring host-defense peptides by showing that skin secretions a frog belonging to the sub-family Leiuperinae contain cytotoxic peptides of a type not previously described. The work has clinical implications in that one particular peptide, pustulosin-3, has therapeutic potential as a template for development into an anti-cancer agent. However, further structure–activity studies are required to design analogs that show greater selectivity for tumor-derived cells compared with non-neoplastic cells.

### Supplementary Information

Below is the link to the electronic supplementary material.

Supplementary file 1: (DOCX 18 KB)

## Data Availability

The data presented in this study are available on request from the corresponding author.
