# Supplementary material for: Purification, conformational analysis and cytotoxic activities of host-defense peptides from the Tungara frog Engystomops pustulosus (Leptodactylidae; Leiuperinae)
Source: Amino Acids. 2023 Aug 7;55(10):1349–59. doi: 10.1007/s00726-023-03312-2 (PMC10689532; doi:10.1007/s00726-023-03312-2)
Supplement: Supplementary file 1 — (DOCX 18 KB) [file 726_2023_3312_MOESM1_ESM.docx]

**Supplementary Material**

*E. pustulosus* 1 F*WKADVKEIGKKLAAKLAEELAKKLGEQ

*E. pustulosus* 2 F*WKADVKEIGKKLAAKLAEELAKKLGEE

*E. pustulosus* 3 D*WKETAKELLKKIGAKVAQVISDKLNPAPQ

*L. ocellatus* 1 G*VVDILKGAGKDLLAHLVGKISEKV^a^

*L. ocellatus* 2 G*VLDIFKDAAKQILAHAAEKQI^a^

*L. ocellatus* 3 G*VLDILKNAAKNILAHAAE*QI^a^

*L. ocellatus* 4 G*LLDFVTGVGKDIFAQLI*KQI^a^

*L. fallax* G*VVDILKGAAKDIAGHLASKVMNKL^a^

*L. laticeps* G*VVDILKGAAKDLAGHLATKVMNKL^a^

*L. pentadactylus* G*LLDTLKGAAKNVVGSLASKVMEKL^a^

*L. syphax* G*VLDILKGAAKDLAGHVATKVINKI^a^

*L. validus* 1 G*VVDILKGAGKDLLAHALSKLSEKV^a^

*L. validus* 2 G*VLDILKGAGKDLLAHALSKISEKV^a^

*L. validus* 3 G*VLDILTGAGKDLLAHALSKLSEKV^a^

*L. latrans* 1 G*VVDILKDTGKKLLSHLMEKIG

L. latrans 2 G*VVDILKDTGKKLLSHLMEKVG

*L. latrans* 3 G*VLDIFKDTGKKLLSHLMEKVG

*L. latrans* 4 G*LLDFLKAAGKGLVSNLLEKVG

L. latrans 5 G*LLDFLKAAGKGLVSNLIEKVG

*L. latrans* 6 G*VLDIFKDAAKQILAHAAEKIG

*L. pustulatus* 1 G*VFDIIKDAGKQLVAHAMGKIAEKV^a^

*L. pustulatus* 2 G*VFDIIKDAGKQLVAHATGKIAEKV^a^

*L. pustulatus* 3 G*VIDIIKGAGKDLIAHAIGKLAEKV^a^

*L. pustulatus* 4 G*VFDIIKGAGKQLIAHAMGKIAEKV^a^

*L. pustulatus* 5 G*VFDIIKDAGRQLVAHAMGKIAEKV^a^

*L. pustulatus* 6 G*VFDIIKGAGKQLIAHAMEKIAEKVGLNKDGN

*L. pustulatus* 7 G*VFDIIKGAGKQLIAHAMGKIAEKVGLNKDGN

*L. pustulatus* 8 G*VFDIIKGAGKQLIARAMGKIAEKVGLNKDGN

*L. insularum* 1 G*LLDLLKGAGKGLLTHLAS*QI^a^

*L. insularum* 2 G*LLDFFKGAGKELLTHLAS*QI^a^

*L. insularum* 3 G*VIDILKSLGKNILTNLASKLSDNTA

*L. nesiotus* 1 GAVVDILKGAGKNLLSLALNKLSEKV^a^

*L. nesiotus* 2 GAVVDILKDTGKNLLSLALNKLSEKV^a^

*L. nesiotus* 3 G*IFDVLKNLAKGVITSLAS^a^

*L. nesiotus* 4 G*LFDVLKNLAKGVITSLAS^a^

Supplementary Figure 1. A comparison of the primary structures of the pustulosins from *E.* *pustulosus* with ocellatins from frogs from a range of species belonging to the sub-family Leptodactylinae. Amino acid residues that have been strongly conserved between the pustulosins and the ocellatins are shown in red. Gaps denoted by * are inserted into the sequences to maximize sequence similarity.
